# Oral Delivery of Biologics via the Intestine

**DOI:** 10.3390/pharmaceutics13010018

**Published:** 2020-12-24

**Authors:** Roger New

**Affiliations:** 1 Proxima Concepts Ltd., London NW1 0NH, UK; rogernew@proximaconcepts.com; 2 Faculty of Science and Technology, Middlesex University, London NW4 4BT, UK

**Keywords:** oral delivery, biologics, intestine, receptor-mediated uptake, permeation enhancers, paracellular transport, transcellular transport

## Abstract

Biologics are currently one of the most promising avenues for therapeutic interventions in conditions such as metabolic disease, ageing and inflammatory disorders, and for chronic ailments, oral delivery of such medicines has for years been recognised as an important goal. Despite decades of intensive research, oral delivery of biologics is only just starting to prove feasible. There has been much talk about the barriers to uptake of biologics, and indeed, one function of the intestine is to prevent, in one way or another, passage of unwanted materials across the gut, and yet, grams of biological agents both large and small pass across the intestinal cell wall every day. This review first describes the functioning of the gut under normal circumstances, then identifies the principle biological mechanisms which have been harnessed successfully, to date, to achieve oral uptake, outlining the pros and cons of each approach. Examples with different biologics are given, and information on result of the latest clinical trials is provided, where available.

## 1. Introduction

Administration of biologics via the oral route is an exercise undertaken for a variety of different reasons, and, accordingly, a number of different strategies are adopted in order to achieve the desired result. There are different routes into and out of the intestine and the key is to match the former with the latter.

This review focuses on the intestine as the site of activity/uptake, and administration via the “oral route” is taken as occurring as a result of swallowing something which then passes down the oesophagus and through the stomach before entering the intestinal tract, where it exerts its activity. Crossing the stomach is readily achieved by use of pharmaceutical coatings (‘enteric coatings’) which are polymer films sprayed onto the outside of a capsule, tablet or pellet which resists dissolution at low gastric pH, and then dissolves in the raised pH of the lumen of the intestine. Recently, capsules have been created which exhibit these properties, without the use of such coatings, by companies such as Capsugel, ACG and Gelita (see also Barbosa et al. [1]). Interestingly, protection against stomach acidity is more readily achievable in man than animals, since the difference between gastric pH (ranging from 1 to 3) and intestinal pH (5 and above) is greater than other mammals (even primates) where stomach pH often reaches 5 or higher under normal circumstances (see Kararli [2] for a comprehensive review of differences in gastro-intestinal physiology of a range of different animal species).

Under normal circumstances, four things can happen to biologicals (typically polypeptide or proteinaceous materials) when they find themselves in the intestine.

(1). They are broken down into their constituent parts, amino acids principally, which are then taken up by receptor processes across the gut epithelium, as part of the process of digestion, leading to uptake of nutrients.
(2). Certain proteins are resistant to digestion in the gut, and interact with cell receptors, usually with the aim of translocating across the enterocytes in intact form. Examples of such proteins are immunoglobulin (IgG1 and IgA) and intrinsic factor. Other proteins of microbial origin can also resist breakdown in the gut and are able to highjack these endogenous uptake mechanisms and manifest pathogenic effects.
(3). Other proteins, again of microbial origin, having survived attack by proteases, and often still associated with the cells they derive from, are taken up by Peyer’s patches, and stimulate an immune response.
(4). Other proteins resisting degradation are also taken up by Peyer’s patches, whereupon a potential immune response towards them is suppressed.

## 2. Architecture of the Intestinal Tract

### 2.1. Gut Immune System

The immune system of the gut is the largest single immune organ of the body, comprising, as it does, approximately 70–80% of all the body’s immune cells [3]. It is made up of several different components, the most clearly defined of which are the Peyer’s patches, accretions of lymphoid cells comprising lymphocytes, macrophages, and dendritic cells assembled under a layer of M cells interfacing with the lumen of the gut, whose task is to take up potential antigens from the lumen and pass them to antigen presenting cells. Other parts of the intestine also play a role, particularly the lamina propria, a compartment stretching the length of the intestine basal to the enterocytes, feeding into the intestinal lymphatics, and a population of intra-epithelial lymphocytes (IEL) embedded within the tight junctions of the much larger epithelial cells. For a general review of formulations for oral vaccines, see New [4].

The “default setting” of the gut immune system, in the absence of any powerful immuno-stimulant, is to down-regulate (i.e., suppress) any potential immune response to any proteins encountered (particularly in the diet) and it is probably the intra-epithelial lymphocytes which play a defining role in this respect, since IEL comprise a large population of IL10-secreting Tγδ cells [5,6], which are implicated in the maintenance of regulatory T cells, whose job is to curb the excesses of the immune system. Any formulation administered to the intestine, therefore, needs to be one that allows this population of cells to fulfil its function undisturbed to avoid the risk of inducing food allergies or inflammatory disease. The good news is that, as long as there is no immunostimulant incorporated in such a formulation, it is likely that the gut immune system, if it were to recognise a biologic incorporated therein, would act to suppress a potential immune response towards it, even if it were nit of human origin.

The M cells of Peyer’s patches have a propensity to take up materials in particulate form, particularly particles of a size range in the order of 0.1 microns in diameter [7], and preferably with hydrophobic surface characteristics. Suggestions have been made in the past that Peyer’s patches may be a promising route for delivery of biologics into the rest of the body after oral administration [8]. While entry of particles into these structures is well documented, it seems unlikely that throughput will be sufficiently rapid to permit significant quantities to enter the draining lymphatics or bloodstream via this route. The M cells constitute a very small proportion of the total surface area of the intestine, so it is to be expected that the most promising routes across the gut wall will be via those cells comprising the vast majority of intestinal tissue, namely the intestinal epithelial cells, or enterocytes.

### 2.2. Components of the Digestive System

**Figure 1:**
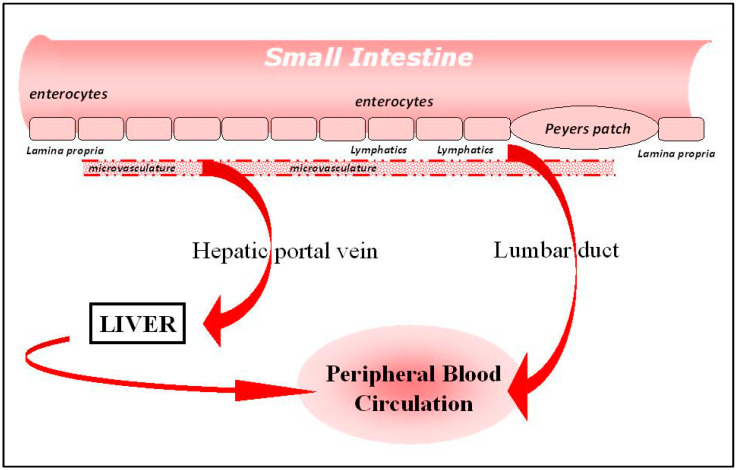
Architecture of the intestine.

Before describing how transport across these cells can be brought about, it is important to be aware of the different sites in the body at a biologic can end up, and this is related to the way in which the agents to be delivered interact with the specific architecture of the intestine. A diagram of the intestine is shown in Figure 1, in which it can be seen that materials crossing the intestinal cell barrier can follow one of two pathways to enter the rest of the body.

On the one hand, the agent arriving in the fluid basal to the enterocytes can diffuse rapidly into the microvasculature draining the whole length of the intestine, and then pass, via the splanchic vein into the portal vein, and then to the liver. Unlike small-molecule drugs, large biologics are unable to enter the liver cells unaided, and will quickly flow through the liver and into the peripheral circulation, mixing into the central blood pool of the body. One exception is when receptors for the biologic are to be found on the surface of the liver cells themselves, and this situation will be discussed in more detail later. It is important to note that, surprisingly, there is no direct access into the peripheral circulation at all for materials absorbed from the intestinal tract. There is, however, an indirect route, described in the next paragraph.

As mentioned above, there is a fluid compartment lining the entirety of the intestinal tract, namely the intestinal lymphatics, a network of channels, membranes and valves which collect fluid from the lamina propria and allow it to pass, by virtue of the action of peristaltic and other semi-autonomous motions of tissues in the abdomen, along its length until it reaches a junction in the lymphatics known as the lumbar duct, which interfaces with the bloodstream, and allows material to enter into the peripheral circulation without passing through the liver.

Which of these two routes an agent takes, after crossing the gut wall, is determined to a large extent by the physical properties of the molecule in question. Small hydrophilic proteins will diffuse rapidly across the vascular endothelium into the intestinal microvasculature, and proceed quickly into the hepatic portal vein. Larger molecules, particularly lipophilic agents, or those associated with lipids and/or in particulate form, will diffuse less readily across the microvasculature, and, after a certain time delay, will arrive at the lumbar duct and enter the central blood pool.

For any protein in the lumen of the gut, the barriers to entering the bloodstream, whether via the portal circulation, or along the lymphatic channels, are 3-fold.

1. Breakdown by intestinal proteases
2. Impairment of diffusion by the mucin layer
3. The monolayer of intestinal cells forming the wall of the intestine, which are impermeable to large molecules.

One thing to be aware of is the influence of the intestinal environment on all of the processes described above, in terms of pH, salt concentration, surfactant content, etc. This can vary depending on whether the digestive system is in the fed or fasted state. In vitro models should be conducted in simulated intestinal fluid (SIF, quite different from simulated gastric fluid), and recipes for SIF in both the fed and fasted states have been established by Dressman and Reppas [9], which not only mirror pH (5 in the fed state, 6.5 in fasted) of the lumen, but its natural buffering capacity as well.

#### 2.2.1. Intestinal Proteases

Four different enzymes are secreted into the intestine by the pancreas-trypsin, chymotrypsin, elastase and carboxypeptidase. Trypsin and chymotrypsin are present in the largest quantities, and all these proteases are secreted in the form of inactive pro-enzymes (zymogens). Activation of the proenzymes is brought about by cleavage of N-terminal fragments by trypsin, and nascent trypsin precursor (trypsinogen) is itself cleaved when it encounters, in the intestine, active trypsin which has been cleaved and activated at an earlier stage. There is also thought to be a membrane-associated protease in the duodenum also convert trypsinogen to trypsin. Properties and characteristics of the different enzymes are shown in Table 1.

**Figure 2:**
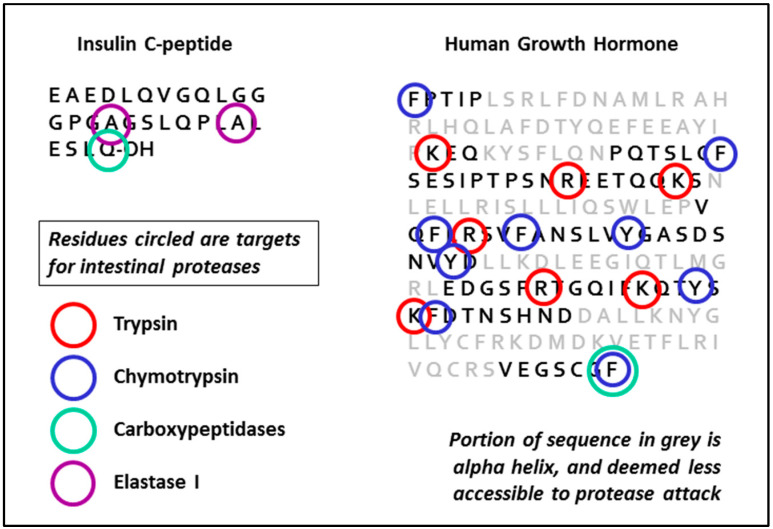
Vulnerability of peptide sequences to degradation by gut proteases.

A number of natural inhibitors are given in the table, some of which are either GRAS-listed or pharmacopoeial. Other inhibitors derived from non-metabolisable peptides (e.g., comprising D-amino acids) matching the appropriate specificity of each enzyme also exist, although none are currently approved as pharmaceuticals. The same goes for non-specific inhibitors (e.g., PMSF) which are highly potent, but toxic. The routes and extent of potential breakdown for two representative biologics is shown in Figure 2, derived as a logical conclusion of the information in Table 1 (see also Wang et al. [10]).

#### 2.2.2. Mucin

All mucosal surfaces in the body are covered by a protective layer of high molecular weight proteins known as mucins. In the intestine, mucin is secreted by goblet cells, and is present as a thick layer throughout the length of the digestive tract with the exception of the M cells covering the Peyer’s patches.

Each mucosal surface has its own version of mucin (for a general review, see Bansil and Turner [11]), and for the intestine the predominant variant is MUC2. (Mucin in the stomach is MUC6 and in the oral cavity and vagina is MUC5B; the lung contains a mixture of all of these mucins, and many others). Intestinal MUC2 is a large protein consisting of long stretches of random coil peptide chain, comprising almost entirely threonine and serine, linking globular domains which can form non-covalent and covalent intermolecular bonds with similar domains on other mucin molecules. There are at least four types of such binding interactions which mucin domains can take part in. Portions of the mucin molecule are highly glycosylated on asparagine residues, and hydrogen bonding between hydroxyl groups on sugars on adjacent molecules can take place very readily. Other areas are rich in hydrophobic residues which interact with each other to minimise contact with water, and salt bridges between arginine and carboxyl side chain residues are also a feature. Finally, an important mechanism of crosslinking is through formation of covalent bonds resulting from interaction of cysteines in adjacent mucin molecules to form disulphide bridges. Indeed, whereas in most proteins the number of cysteines can be counted in single digits, in mucin there are stretches of peptide in which cysteines comprise 10% of the chain. Intermolecular interactions of the type described above give rise to a cohesive lattice of mucin, which is resistant to physical removal, and which constitutes a barrier to the diffusion of large molecules across it.

Much work has been conducted to find ways of breaking down the mucin layer, particularly in the lung, where overproduction causes problems in conditions such as cystic fibrosis. Agents which have been successful in helping to solubilise mucin include acetyl cysteine, which presumably acts to break disulphide bridges by reduction and/or reforming of the bridge with acetyl cysteine. Unfortunately, such reactions occur most readily at neutral pH, so to date, use of such molecules to enhance transport across the intestinal mucin barrier in vivo, where the pH is significantly below neutral, have not been reported.

**Figure 3:**
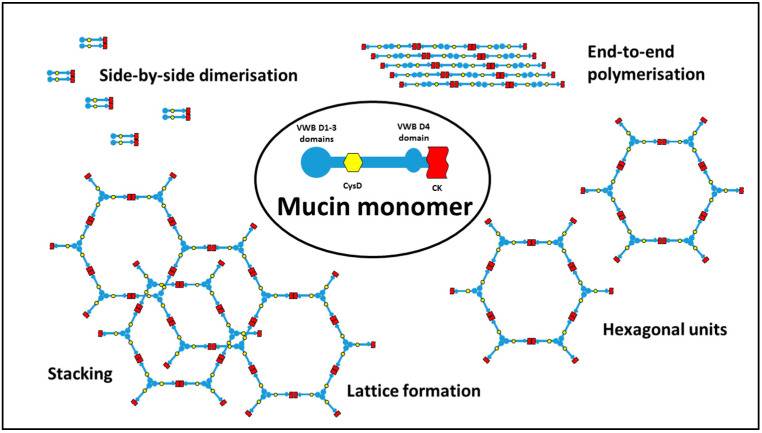
Potential configurations adopted by mucin molecules. VWB—domains possessing strong homology with domains in von Willebrand factor; CysD—cystine disulphide-rich domain; CK—cystine knot domain.

Interestingly, MUC2 bears a lot of similarity to von Willebrand factor (VWB), involved in the blood clotting cascade, and forming a cross-linked polymer network as part of the clotting process. Both MUC2 and VWB have terminal domains which interact via covalent S-H bonding to form beads with 2-fold of 3-fold symmetry, depending on the conditions. As the mucin is secreted from the goblet cells, a complex process ensues to give preference to end-to-end multi-monomer polymerisation, rather than limited side-by-side dimerisation. As the mucin continues to spread over the surface, one proposal is that mucin forms a hexagonal lattice, with the beads at the vertices of the hexagons, which can then stack on top of each other, fixed via non-covalent interaction of the CysD regions, to give an extensive, rigid 3-D network capable of forming a barrier to passage of macromolecules. Some of the potential configurations which could be adopted are shown in Figure 3. For detailed descriptions of mucin structure, see discussions by Ambort [12], Nilsson [13] and Javitt [14].

#### 2.2.3. Intestinal Cell Barrier

##### Intestinal Cell Dynamics

**Figure 4:**
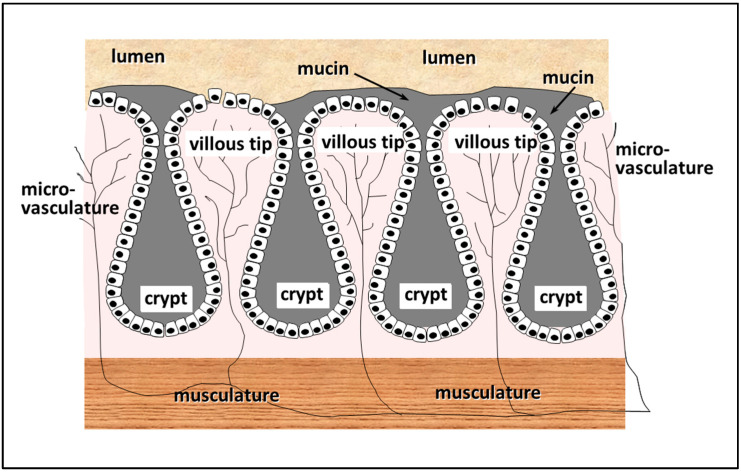
Schematic image of the mucosa of the small intestine.

The internal surface of the intestine is a continuous layer of cells, covered, for the most part, by mucus, the function of which is to lubricate the surface of the intestinal wall, and protect the cells from physical damage due to shearing stresses, etc. The layer of intestinal cells itself is not planar, but adopts a villous configuration, so that the inner surface of the intestine is composed entirely of long columnar processes approximately 50 cell widths in height, the tips of which come into direct contact with the luminal contents, while the crypts at the base in between the villi create a more sequestered environment (see Figure 4). Microvasculature is present along the entire length of each of the villi, so that cells at the tips, as elsewhere, have direct access to blood micronutrients required for normal function. Within the central core of the villi, a region known as the *lamina propria* is to be found, which is populated by a significant proportion of lymphoid cells. Interstitial fluid basal to the enterocytes is drawn through the lacteals to lymph ducts and drains into the blood stream via the lumbar duct. Beneath the crypts, a layer of musculature is located, encircling the whole of the outer surface of the gut, which acts as a physical barrier, and is also responsible for moving material along the gut by peristalsis, and for maintaining lymphatic flow. A comprehensive overview of gut mucosal morphology is given in in Madara et al. [15].

The epithelial cells of the small intestine probably represent the most dynamic cell population in the whole body. Cells in the crypts of the microvilli are constantly dividing, and migrate up to the tips of the microvilli, differentiating in the process, until they are shed from the tips into the lumen, to be replaced by the next generation of migrating cells. In the rat, migration of cells from the crypt to the tip takes on average two days, 2% of the total cell population being shed per hour [16]. However, since all the cells shed are sloughed off from the tips of the villi, and since the tips comprise 2% of the total length of the villi, it can be concluded that the entire cell surface in contact with the lumen of the intestine regenerates every hour.

##### Intestinal Permeability under Natural Circumstances

The renewal of the gut surface clearly involves dynamic dissociation of cells from each other. Mechanisms have been put forward, based on in vitro studies, suggesting ways in which cells can be extruded from monolayers without breaching integrity. Under the more dynamic conditions in vivo, however, it is likely that aqueous channels will be formed, allowing passage of fluid and solutes in either direction across the gut wall. Studies looking at transport of macromolecules across intact gut indicate that this is indeed the natural state of things. It is estimated that, in a normal intestine, approximately 200 mg of solid material pass out of the intestine into the rest of the body via this route over a six hour period [17]. Over a single day, or over a lifetime, this represents a not inconsiderable amount of material, which the body is undoubtedly able to cope with, without untoward effects. A study performed in humans [18] showed that 40 mg of PEG3350 was excreted in the urine of 24 h after ingestion of 40 g orally, demonstrating that significant quantities of high molecular weight material are capable of entering the bloodstream from the gut on a continuous basis under normal circumstances. Similar conclusion may be drawn from work with inulin [19], a polysaccharide of molecular weight 5200, very similar in size to insulin.

Many agents are already known to augment this process still further. Thioates, found in foods such as onions and garlic, are known to permeabilise the gut, and aspirin, one of the most widely used medications worldwide, increases the intestinal permeability by an order of magnitude every time it is ingested [20]. The effect is thought to be mediated via uncoupling of mitochondrial oxidation, and occurs without stimulation of any inflammatory processes in the small intestine [21]. A recent study in rats [22] showed that the hydrolytic products of food digestion themselves are responsible for perturbing the epithelial lining of the villous tips (but not elsewhere), and concluded that “... the intestinal epithelium is injured and restitutes during the normal course of digestion and absorption of a meal.” Another study [23] looking directly at the effects of deoxycholate show that effects on the epithelial lining are restricted to the villous tips, and that restoration of epithelial continuity is complete fifteen minutes after cessation of exposure.

In all cases, adverse effects due to material leaking from the intestine have never been reported, even though this undoubtedly happens on a regular basis. A number of physiological conditions exist where the intestinal wall presents even less of a barrier to passage of luminal contents. In most mammalian species, including man, neonates are born with intestines in which tight junctions between cells are permanently open, and it is several weeks before ‘closure’ takes place, reducing the opportunity for intestinal contents to pass out. Protection of infants is said to be afforded by antibodies in mothers’ milk, in the absence of a mature immune system in the neonate. The ability of the body to function normally, even with an intestine whose integrity is markedly reduced, suggests that manipulation of intestinal permeability over a short time scale is not a procedure prejudicial to normal health.

##### Abnormal Physiology

A number of situations occur in which the intestinal permeability is chronically elevated during adult life, for example in pathological conditions such as Crohn’s disease. This is a disease principally affecting the right ascending colon, although often with small bowel involvement. It is now generally recognised to be due to both genetic and environmental factors [24,25], triggered by prolonged exposure to pathogens [26] such as intra-cellular parasites, e.g., *mycobacterium avium subsp. paratuberculosis*. Tight junctions between all epithelial cells are opened in response to elevated levels of inflammatory mediators and the standard treatment for the disease is administration of anti-inflammatory agents. In spite of leakage of large quantities of intestinal material in all patients, only 20% display extra-intestinal manifestations and no acute symptoms such as fever, shock or septicaemia are seen. Of the extra-intestinal manifestations reported, these fall into two categories—hepatic and bile duct-related, due to a reduction in the size of the recirculating bile acid pool, and immunological/inflammatory (e.g., rheumatoid arthritis), probably an extension of immune manifestations in the gut.

Many approaches to oral delivery involve the use of ‘permeation enhancers’—a catch-all term used to describe any chemical that increases passage of materials across the gut wall. However, the events accompanying topical exposure of the lumenal surface of the gut to permeation enhancers, for delivery of peptides or proteins, are markedly different from those involved in causing and maintaining the pathology of inflammatory bowel diseases. In the latter, a prolonged microbial assault (probably via the colon) brings inflammatory triggers into contact with immune cells in the *lamina propria*, resulting in generation of a chronic local inflammatory response. In sharp contrast, permeation enhancers applied topically have little opportunity to contact any epithelial cells other than those at the villous tips, since a thick mucus layer fills the entire volume between adjacent villi. Cells at the tips of the villi are perturbed transiently, allowing passage of proteins directly into the microvasculature draining these cells. Material entering via this route has little opportunity to persist in the *lamina propria*, since it is removed rapidly by the microvasculature, whose permeability is heightened in this region.

Methods for traversing the layer of cells separating the lumen of the gut are the major factors which distinguish the various approaches to enhancing oral delivery across the gut, and the rest of this review will accordingly focus on this key aspect.

##### Tight Junctions

**Figure 5:**
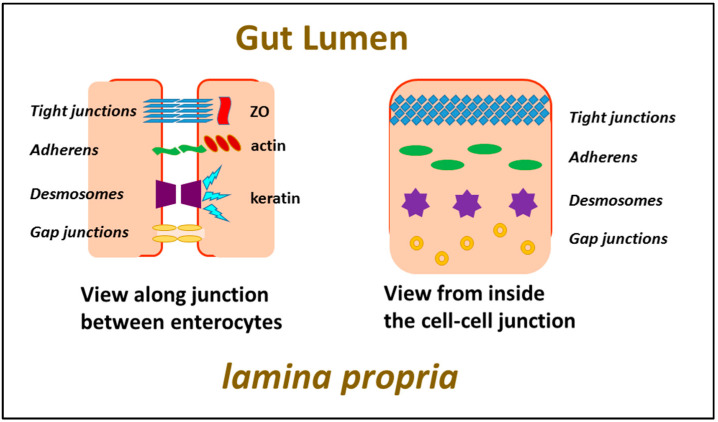
Intercellular interactions of enterocytes.

Enterocytes form a relatively impenetrable barrier to passage of material across the monolayer by virtue of the fact that cells abutting each other are held together very closely, with a continuous seal existing at all sites along the membrane interface of adjacent cells as a result of intercellular binding of proteins in the two membranes which form what are known as tight junctions. The proteins responsible for this are transmembrane proteins, linking the cell exterior with the cytoskeleton lying underneath the cell membrane. Other intercellular attachments also exist, mediated by adherens proteins and desmosomes, whose purpose is to maintain physical integrity of the cell layer, rather than a barrier to solutes (see Figure 5).

Although as many as forty different proteins have been identified as residing within tight junctions and having a role there, it is the claudins which appear to be responsible for maintaining their physical integrity. There are many different iso-forms of claudins, and claudin-9 is the one playing a role in tight junctions in the intestine. Its transmembrane portions consist of four alpha-helices, with a C-terminal intra-cellular domain possessing a PDZ binding site permitting attachment to the cytoskeleton. The extra-cellular portion contains a beta-pleated sheet which acts as a cation-sensitive ion pore. In order to maintain the physical barrier of the tight junction, extensive intermolecular interactions are formed when the claudin molecules align alongside each other in the membrane (“cis” interactions) as well as bridging the gap between cells to enter into “trans” interactions with claudins in the opposing membrane. Disruption of these interactions can occur when molecules such as the C-terminal domain of *C. perfringens* toxin bind to the extra-cellular domain of claudin-9. Other proteins are also thought to play a role in stabilising tight junctions, namely occludin, tricellulin and JAMA-1, and these can also act as targets to aid in opening of tight junctions.

##### Transcellular Transport Across Intestinal Cells

**Figure 6:**
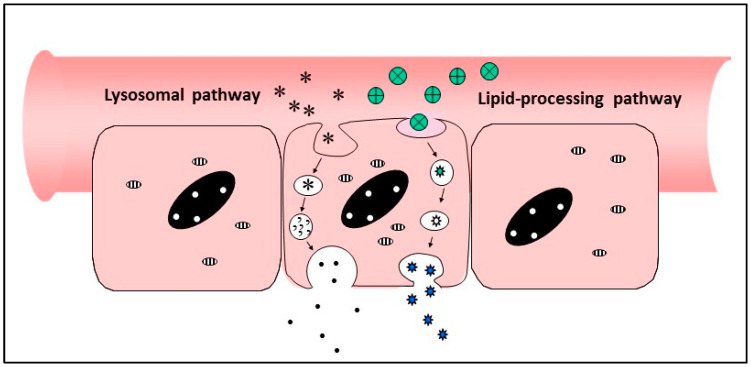
Transcellular passage of materials across the gut wall.

An alternative route to paracellular transport in between intestinal cells is transcellular transport directly into and across the enterocytes themselves (Figure 6). Although this seems even more of a tall order than opening of the tight junctions, it should be remembered that transcellular passage is occurring naturally all the time, as a method of transferring dietary breakdown products into the bloodstream, and the challenge is to harness these mechanisms for transport of large biologics, rather than just that of nutrients such as amino acids, sugars and lipids.

The pathways to be targeted fall into three different categories—(i) endocytic pathways involving uptake of partially digested materials into endosomes via pinocytosis or vacuolation, accompanied by continues breakdown through enzymic attack as the vacuoles cross the cell and exocytose their contents at the basal face of the cells; (ii) lipid-processing pathways in which fats are taken up in micelles, broken down by lipases and transformed into chylomicrons for secretion into the intestinal lymphatics; (iii) receptor-mediated processes specific for ligands which bind and internalise individual molecules at the apical surface of the enterocyte, followed by transportation across the cell.

## 3. Pharmaceutical Approaches to Delivery—Intestinal Transport by Design

Formulation approaches to getting materials across the intestinal barriers fall into one of three categories, each using the whole range of different vehicles and formulation techniques at one’s disposal—microparticles, emulsions, polymers, permeation enhancers, etc. These different approaches may be thought of as being receptor specific, cell specific or tissue specific, and are dealt with in turn below.

### 3.1. Receptor-Specific Approaches—Biological Methods of Entry

In this strategy, mechanisms employed by the body to allow passage of specific molecules across the intestinal wall are commandeered to function instead with a desired therapeutic payload of interest.

#### 3.1.1. Vitamin B_12_/Intrinsic Factor

This is the archetypal model for receptor-mediated transport of macromolecules across the gut, since intrinsic factor itself is a large protein which is internalised by enterocytes, so the task is simply that of attaching another protein to the Vitamin B_12_ which binds to intrinsic factor, and every other part of the transcellular pathway should already be in place.

Intrinsic factor (IF) binds to Vitamin B_12_ (also known as cobalamin) in the duodenum, and transports it to a receptor on the membrane of epithelial cells in the ileum, known as cubilin. Intrinsic factor is a ~60 kD protein consisting of alpha and beta domains joined by a flexible linker. When IF binds to cobalamin, it sandwiches the vitamin between its two domains, which come together to form a new combined epitope which is recognised by cubilin. Binding of cubilin to the modified IF results in its internalisation with the cell, in conjunction with another membrane protein, amnionless. Cobalamin has a larger number of functional groups through which it can be conjugated to drugs and proteins. While many of these are hidden when the vitamin is entrapped inside the cleft between the alpha and beta domains, there are parts of the molecule which remain exposed in the IF/cobalamin complex, notably the nucleoside hydroxyl group, through which biologics can be attached without interfering either with the binding of the cobalamin to IF, or binding of the IF/cobalamin complex to cubilin, and subsequent internalisation. Once taken in to the lysosomal vacuole, the IF is degraded by cathepsin L (a cysteine protease with specificity for aromatic residues), and transferred to transcobalamin (TCII) which mediates its exocytosis. Fortunately, the cobalamin binds to TCII in a very similar way to IF, to agents can be conjugated to the cobalamin in such a way that they do not interfere with this transfer process. It should be mentioned, however, that the inside of the vacuole is a proteolytic environment, so checks need to be made that the biologic to be delivered is not susceptible to the action of cathepsins. It is also worth noting that the vitamin remains bound to TCII once it is in the bloodstream, so measure may need to be taken to dissociate the biologic from the cobalamin before the TCII is internalised by its target cell.

Much of the early work on application of Vitamin B_12_ transport to delivery of biologics was conducted by the group of Russell-Jones, who demonstrated uptake of molecules like GCRF, EPO [27] and LHRH antagonists [28]. It was clear, however, that the system was limited by the fact that the cubilin receptor density in the ileum was sufficient only to allow uptake of ~2 ug of Vitamin B_12_ per day—equivalent to 100 ug of peptide or protein at the most, depending on its molecular weight. To overcome this, conjugation of Vitamin B_12_ was investigated, not directly to the protein, but to microparticles containing the protein interest instead, thus increasing the protein:ligand ratio many-fold, and also protecting the protein from attack by protease in the lumen of the intestine. In this way, success was achieved with insulin encapsulated in dextran nanoparticles 150–300 nm [29], where, in streptozotocin-treated rats, at least 5 ug was absorbed from the gut, achieving a fall in plasma glucose of 70%, thus demonstrating that it was in biologically active form. While potentially a very promising avenue for delivery, it is not clear whether sufficiently insulin can be administered in an economically viable fashion, since, while overall bioavailability appeared to be good ~30%, there may be manufacturing challenges to overcome, particularly in conduct and characterisation of the chemical processes involved. The technique could find application with peptides of higher potency that insulin, where the quantities with need to be absorbed are smaller, and where day-to-day variations in level of Vitamin B12 receptors is not a concern. For a general review of delivery of both small and large molecules with Vitamin B_12_, see Clardy et al. [30].

#### 3.1.2. Biotin

Another intestinal receptor which has been targeted as a route for delivery of biologics is that for biotin. Like Vitamin B_12_, biotin is an essential pre-requisite for the functioning of many biochemical processes in the body, but is not synthesised by mammals, so is either taken up from the diet, or manufactured by commensal bacteria in the large intestine. In either case, the receptor involved is the sodium-dependent multivitamin transporter (SMVT), which is found in the apical membranes of enterocytes, as well as hepatocytes, among other cell types. An insulin delivery system devised by Geho et al. [31,32] has been described, in which biotin acts as a targeting agent to carry insulin both across the intestinal cell wall, and to the liver cells themselves, where the insulin exerts its primary activity in glucose regulation. The formulation is a liposome-like particle, although it is not clear whether the insulin is encapsulated with the phospholipid envelope, or bound/cross-linked to the outside. A very high degree of efficacy was reported, both in animals and in humans, implying a percentage uptake of 50% or greater, but the exact degree of bioavailability is difficult to assess, since in some or all of the oral formulations employed there were, in addition to insulin itself, chromium ions and biotin, both of which are known to potentiate the activity of insulin in glucose regulation.

It is also difficult to understand the precise mechanism by which the insulin/biotin complex is taken up, since, in contrast to Vitamin B_12_, conjugation of agents to biotin via its free carboxyl group actually interfere with binding and internalisation of biotin by enterocytes. Presumably the uptake mechanism involves a generic vacuolation process as a result of crosslinking of the SMVT on the membrane, followed by exocytosis after transport of the vacuole across the enterocyte. The way in which the insulin-coated particle interacts with receptors on the surface of hepatocytes is also a mystery.

Of greater concern, however, is that, in addition to targeting to hepatocytes, biotin has a propensity for tumours, where the biotin receptor is up-regulated, and in particular for nascent tumour cells. Targeting of insulin, a known growth factor, the poses the risk of encouraging tumorigenesis, and this may be the reason that the promising results were not followed up. In order to overcome this possibility, a mechanism for rapid dissociation of the carrier from biotin in the bloodstream would have to be engineered. It could be, however, that the system as is may be of value for delivery of other agents via the oral route—in particular cancer therapeutics directed towards the liver. It is also worth noting that, in addition to biotin, the same SMVT receptor can act as a transporter for other lipidic moieties such as pantothenic acid and lipoic acid, so it is possible that these agents could also be used as targeting ligands to cross the gut wall.

#### 3.1.3. Bile Salts

In contrast to biotin and Vitamin B_12_, the transport of bile salts across enterocytes occurs continuously and at a very high rate. In an adult, the average quantity absorbed in a day is 18–20 g [33]. This uptake occurs mainly in the ileum, and is part of the enterohepatic recycling process whereby the bile salts, having fulfilled their task of solubilising fats and lipids in the diet, are taken across the gut wall into the portal system where they pass rapidly into the liver to be channelled into the gall bladder ready for secretion into the intestine again to aid the next round of the digestion process.

The receptor on the apical surface of enterocytes is the Apical Sodium-dependent Bile acid Transporter (ASBT) [34,35], and the bile salts are secreted at the basolateral surface by OST. ASBT has a broad specificity for all common bile salts, and it appears that small drug molecules can be linked to either the 3-OH position or via the carboxyl group at C-24 without jeopardising binding or uptake [36]. This can apply to peptides as well, providing they are very short and lipidic [37]. In general, however, larger, more hydrophilic peptides are not taken up, even if they are able to bind to the receptor. One may speculate that internalisation of the bile salt is mediated not by a vacuolation process, like Vitamin B_12_, but via a flip-flop mechanism across the membrane, which is inhibited sterically by conjugation with large molecules. One report of successful uptake of a large peptide, however, has been provided by Morrison et al. [38], who attached gastrin-34 to cholic acid at the C24 position via an amide linkage, and showed biological activity of the peptide in rats in vivo. The peptide dose was quite high, however, and it is not clear whether the percentage taken up was sufficient to yield a viable product commercially.

One further issue that would need to be addressed would be a way of separating the bile salt rapidly from the peptide, since ASBT is also found on hepatocytes, so the peptide could get diverted to the gall bladder if the two are not separated.

#### 3.1.4. Transferrin

Transferrin is a large glycoprotein (MWt 79.5 kD) which is internalised by many types of cell through receptor-mediated endocytosis, facilitating the uptake of iron into tissues for use in intra-cellular biochemical processes. There are two different receptors for transferrin, TfR1 and TfR2 (high and low affinity, respectively), and these have been targeted with transferrin fusion proteins as a means of introducing therapeutic biologicals into or through cells. The receptors are over-expressed in certain types of tumour cell, and the ability of the receptor to allow transport of transferrin-antibody fusion proteins across the blood-brain barrier has been described [39].

There are conflicting reports regarding the presence of transferrin receptors in the intestine, with some authors suggesting that it is found only on the basolateral surface of enterocytes, and not apical. The receptor may be up-regulated or relocated in IBD, and if transferrin does play a role in intestinal tissue, it seems, in contrast to other parts of the body, not to be involved in iron uptake, but rather to help regulate intestinal immune tolerance [40].

Whatever its intended role in the intestine in nature, transferring has been investigated as a potential carrier for therapeutic biologicals, in the form of fusion proteins with insulin [41], pro-insulin [42] or human growth hormone (GH) [43], all in rats. In the latter study, GH retained its activity in the fusion protein providing it was linked to transferrin via a long 50-amino acid helical spacer. After oral administration, a modest body weight gain in hypophysectomised rats was observed, suggesting that approximately 2% of the protein was taken across into the bloodstream. The authors surmised that the growth hormone was being broken down by intestinal proteases (trypsin and chymotrypsin) and higher levels of delivery could be achieved in the presence of protease inhibitors.

#### 3.1.5. Cholix

Cholix is an exotoxin from *Vibrio cholera* which binds to the lectin mannose-binding protein 1 (LMAN-1) on the surface of enterocytes, and is being promoted as a means of uptake of siRNA and proteins by Applied Molecular Transport in US. Fusion proteins comprising a truncated, non-toxic domain of Cholix which still retains its ligand-binding activity have been shown to be translocated across cells from apical to basal surface through Rab7^+^/Rab11^+^ vesicles. Transport of hGH has been demonstrated, both in terms of histologic localisation, and observation of an increase in IGF-1 in hypophysectomised mice, but no indication of the efficiency of uptake is available yet [44].

#### 3.1.6. Immunoglobulin Fc

There are a number of different Fc receptors in the body whose principle function is to assist immune cells such as B cells and dendritic cells to present antigens by fixing immunoglobulin molecules on their surface. These receptors belong to the Ig superfamily, with a molecular weight between 50 and 80 kD; in humans the receptor for IgG is coded for by genes located on chromosome 1. There is, however, another receptor for Fc, whose primary function is to facilitate transport of IgG across polarised cells such as kidney, placenta and intestinal cells. It is of a completely different lineage from other Fc receptors, belonging to the MHC family, with a molecular weight of 41 kD, and located on chromosome 19 in humans. It was originally isolated from neonatal intestine in rats, where, it performs the function of transferring maternal IgG across the intestinal wall into the bloodstream, as part of the process of conferring immunity in newborns through ingestion of mother’s milk. The receptor was thus called the neonatal receptor (FcRn) although in fact it is also widespread in the body in adults [45].

The FcRn sits in the apical membrane of enterocytes, in association with beta-2 microglobulin, and two molecules of the receptor bind to the Fc portion of IgG, resulting in its internalisation in endocytotic vesicles, transporting it to the basolateral surface. In some cases, passage of IgG can take place in both directions, leading to a recycling process, in which antigen in the lumen of the gut is transferred to the *lamina propria*, to assist in generation of a systemic immune response. Expression of the receptor on intestinal cells is subject to influence of agents such as TNF alpha or gamma interferon, leading to up- or down-regulation, respectively [46], but the receptor is always present in significant quantities, and in general milligram quantities of IgG can be transferred from the lumen in a day in normal individuals.

One of the first demonstrations of the use of this receptor for transport of biologics was provided by Low et al. [47], who created fusion proteins of the Fc fragment with alpha and beta chains of FSH. Biologically active protein was absorbed into the bloodstream, as shown by increase in weight of ovaries/testes in rats, and inhibin levels in primates. Relative bioavailability was not reported, although a major point of interest was a marked increase in half-life of the FSH fusion protein compared with FSH alone (half-life 69 h cf 11.4 h, respectively, in rats; >200 h in primates cf ~24 h for FSH alone). This is because FcRn in the kidney has a resorptive action, which prevents IgG being excreted rapidly into the urine, and keeps it in the bloodstream. This activity also manifests itself with other proteins incorporating the Fc fragment, and is an added benefit of delivering a biologic orally, using FcRn as a transepithelial intestinal transporter.

It is worth mentioning that FcRn also has a binding site for albumin, so should also be able to transport albumin fusion proteins in the same way as with Fc. Such proteins would also benefit from the resorptive action of FcRn in the kidney, since this is partly responsible for the king half-life of albumin in the bloodstream. The disadvantage of this approach, however, is that the size of the biologic is increased, the ratio of payload to carrier is 1:1, and the presence of the carrier (Fc or albumin) fused directly with the biologic may interfere with its activity in some cases. To overcome these drawbacks, incorporation of biologics in nanoparticles has been investigated.

Two studies have demonstrated success in delivering biologics when encapsulated in Fc-coated PLGA nanoparticles. Pridgen et al. looked at insulin [48], while Shi and colleagues [49] studied the effect of exenatide in nanoparticles very similar to those of Pridgen. Both groups saw reductions in blood glucose, indicating that the proteins were taken up intact, and the magnitude of response was consistent with a level of absorption of the order of 10%.

Precise comparisons were difficult, however, because, in both cases, the pharmacodynamics and pharmacokinetics of oral particles and injected free protein were very different. Imaging data from Shi suggested that the particles stayed in the intestine for longer when coated with Fc (~6–12 h) than when not (4–6 h); whether the particles were bound to the apical cell surface, held inside enterocytes, or trapped in the intestinal lymphatics is unclear, and may be a combination of all three. The size of the particles (100 nm) makes it unlikely that they would pass rapidly into the portal vein. Against this, the data from Pridgen showed that the particles released 50% of their insulin in less than two hours, while the maximum fall in glucose for particles was 4 h onwards (exenatide) and 7–10 h (insulin) compared to 1 h for either peptide when injected i.v.

This approach looks promising as a method of delivery for biologicals in general, although is probably not the method of choice for insulin, where avoidance of high blood levels is important, and direct entry into the liver via the portal vein is desirable.

### 3.2. Non-Receptor-Specific Chemical Methods of Entry

A large number of oral delivery systems have been devised which do not depend on specific interactions with membrane transport mechanisms, but which have actions on the environment, or cells (usually enterocytes) as a whole, which bring about bulk changes allowing the biologics to pass across the intestinal barrier. Because these materials lend themselves readily to processing using traditional pharmaceutical methodologies, a number of these have progressed to late-stage clinical trials.

#### 3.2.1. Muco-Adhesion

One approach employed has been to exploit materials which interact not directly with the cells of the intestine, but with the layer of mucus coating the surface, the rationale being that increasing the retention time in the intestine, particularly at sites close to the gut wall, can allow more time for the drug to be released, and subsequently to diffuse across the mucus layer, thus increasing the concentration of therapeutic agent available to cross the cellular barrier.

A number of disparate agents have been described as potential muco-adhesins, in line with the multi-faceted nature of mucin itself, comprising stretches of concentrated negative charge, sugar residues and hydrophobic regions. A good summary of the principles underlying muco-adhesive interactions is provided by Shaikh et al. [50]. A commonly used adhesin is chitosan, a cationic amino-saccharide polymer, which binds to negatively charged surfaces, and which can be formulated as microparticle carriers. Caution with this approach needs to be exercised, however, since chitosan is known to be an excellent immune adjuvant, which is not an issue for use with small-molecule drugs, but is clearly an undesirable property when attempting to deliver biologics. A large number of polysaccharides derived from foodstuffs are muco-adhesive [51], and the mannose-binding tomato lectin, originally proposed as a bioadhesive for intestinal cell membranes [52] also has the ability to bind to mucin.

A calcium phosphate particle developed by Biolaxy is also reported to owe its success to its muco-adhesive properties. Administration of insulin, encapsulated in the particles gave a reduction of glucose in type II patients indicative of a biopotency of between 1 and 5% [53].

Recently, an approach has been developed by Zhou et al. [54], who have constructed nanoparticles from cysteine-derivatised alginate. The free sulphydryl groups enhance adhesiveness to mucin and thereafter assist in opening of tight junctions by actions on F-actin and ZO-1 protein. The particles were used to encapsulate insulin and glucose oxidase (GO), with the intent to achieve glucose-sensitive release of insulin from the polymer through generation of a hypoxic environment by GO in conjunction with 2-nitro-imidazole. While lowering of glucose was seen after oral administration of particles in diabetic rats, the dose of insulin administered was very high (75 iu/kg), and it is not clear whether release of insulin in vivo occurred in a glucose-sensitive fashion.

A basic paradox regarding the muco-adhesive approach for delivery of biologics is that muco-adhesins immobilise the vehicle on the upper surface of the mucin layer, keeping it away from the cell surface, rather than enhancing contact with the enterocytes. Under normal circumstances, diffusion of large molecules unassisted across the mucus layer is very limited. In addition, mucin is continuously sloughed off from the mucus layer, taking the carrier with it, to be replaced by fresh mucin secreted by goblet cells, so retention at the intestinal wall surface may not be as prolonged as intended. In order for the muco-adhesive properties of compounds such as the above to be of value, they would need to be incorporated as additional components into formulations employing other, complementary mechanisms for enhancement of uptake. Examples of this are seen in the next section.

#### 3.2.2. Paracellular Transport

Paracellular transport refers to opening of the tight junctions between enterocytes, allowing the drugs to be delivered along water channels formed, connecting the lumenal and serosal compartments. While a number of agents have been alluded to above (e.g., *C perfringens* toxin) which can open tight junctions by binding specifically to claudin-9, there are many agents which have been extensively researched which act in a much less specific way, Surfactants can interact with phospholipid membranes to increase their permeability, possibly altering calcium levels in the cytoskeleton, thus weakening the interaction between tight junction proteins.

Oramed have conducted a number of clinical trials with a formulation composed of fish oils, composed of highly unsaturated, fluidising fatty acids, which they believe opens tight junctions. They have shown falls in baseline blood glucose in type 1 subjects [55] receiving 8 mg of insulin three times per day. Further studies aimed at progressing this formulation to market are currently underway in type 2 patients.

The high content of highly unsaturated fatty acids could present stability issues in terms of oxidation effects. Encapsulation of hydrophilic proteins in a hydrophobic oil phase is challenging, and the process employed may be similar to that developed by New et al. [56], or Wang et al. [57]. If the insulin remains associated with lipid on its route to the bloodstream, it is possible that it will stay in the lymphatics and enter the peripheral circulation via the lumbar duct, rather than interacting directly with the liver. A not dissimilar approach has been pursued by Chiasma, who have incorporated the short peptide octreotide in an oily surfactant formulation. Biological activity (reduction in IGF-1 levels) has been seen in humans [58], albeit with a very high dose administered—40 mg upwards per day.

A number of other formulations containing surfactants as major components have been tested in clinical trials. Sodium caprate has been known for a long time as a permeation enhancer [59], and was one of the components of the oral insulin formulation originally developed by Merrion, and tested positive in type 2 diabetic subjects by Novo Nordisk. Although the study gave positive findings, Novo’s oral insulin programme was subsequently discontinued, ostensibly on economic grounds.

Another short-chain fatty acid, geranic acid (3,7-dimethyl-2,6-octadienoic acid), with choline as a counter ion (termed ‘CAGE’) in the form of an “ionic liquid”, has used been used as a permeation enhancer, with efficacy in transdermal delivery, as well as oral. Work with insulin [60] has shown good room temperature stability in the dry solid form, although animal experiments were conducted with capsules filled with aqueous solution which is not a viable long-term formulation approach. The authors suggested a bioavailability of 50% or more could be achieved in rats relative to subcutaneous administration, and have shown evidence of paracellular transport across Caco-2 cells. It is not clear to what extent the uptake seen is due specifically to the geranyl content of the formulation, or whether its presentation as an ionic solution (described as a ‘deep eutectic solvent’) is important. For a recent review on ionic liquids and living cells, see Kumari et al. [61]. It remains to be seen whether these good results in small animal studies translate into humans.

Unigene (now Enteris) pioneered a formulation containing acyl carnitines and citric acid (whose role was to maintain intestinal pH at 3, to reduce protease activity). The technology, Peptelligence^TM^ [62], demonstrated significant uptake of calcitonin in phase 2 studies, with a daily dose of 1 mg [63]. However, the capsule administered is large (size 0, containing ~1 g of formulation excipients, and significant gastro-intestinal effects were noted, in both active and placebo, probably due to the citric acid, for which such effects are known.

#### 3.2.3. Transcellular

##### Lipidic Pathways

The behaviour of amphiphiles in the intestine is a very grey area, since in many cases their mechanisms of action does not seem to be restricted just to changing membrane fluidity. Thus, while at lower concentrations sodium caprate opens tight junctions, at higher concentrations it is also capable of assisting passage of biologics directly across the enterocyte plasma membrane. How this happens is not completely clear. One possibility is that non-covalent association of amphiphile with the peptide to be delivered increases the hydrophobicity of the peptide, and enables it to embed itself in the membrane lipid core, before passing via a flip-flop mechanism into the interior of the cell. Certainly, such a mechanism has been proposed for a series of compounds designed by Emisphere to encourage such a process, using their ‘Eligen’ technology [64], employing molecules with acronyms such as SNAC, CNAC and CNAB. These molecules are amphiphiles constructed of a substituted aromatic nucleus conjugated via a peptide link to the omega-terminus of a short-chain fatty acid, in the form of its sodium salt. There are different versions each employed with a different peptide. In the case of semaglutide, tested by Novo Nordisk [65], and containing SNAC as absorption-enhancing agent, an additional uptake mechanism has been proposed, implicating transcellular uptake in the stomach, brought about by changing the pH or the local microenvironment [66]. Comparison of biological efficacy with a subcutaneous control suggests a level of uptake between 1 and 2.5% [67]. This formulation has already been approved by the FDA.

Another company, Arisgen, has created amphiphiles in the form of crown ethers, which adhere to the polar surfaces of proteins to increase their hydrophobicity [68]. Oral/buccal delivery of leuprolide and exenatide are projects being pursued.

A similar result is probably achieved by Biocon, using a technology originally developed by Nobex, and employed most recently for the delivery of insulin. Here, the amphiphilic molecules are associated with the protein by direct chemical conjugation. In early patents the attachments were short-chain fatty acids (linked to lysine side-chains) with a PEG chain at the omega terminus. In later patents, however, the lipidic and polar portions are switched round, so that the protein is linked to a long hydrocarbon chain via a PEG spacer. In addition to protecting the protein against protease attack, the lipid chains can encourage interaction with the lipid portion of the cell membrane. It is possible that the covalently-attached hydrocarbon chains can also draw the protein through the lipid-processing pathway via fatty acid receptors, leading to vesicular transport across the cell, and secretion with chylomicrons.

In preclinical and human studies, the insulin is taken up very rapidly. A large peak is seen in the serum, raising the question as to whether binding to insulin receptors is reduced because of the chemical modifications. The product is being positioned as a prandial insulin to counteract glucose peaks at meal times, rather than having a longer-term action on basal levels [69]. As a modified insulin, the active is a new chemical entity, subject to stringent regulatory requirements, but clinical trials so far have shown no safety issues.

The lipid-processing pathway may be the target of other types of vehicle, such as the cholestosome [WO2014152795A2], whose object is to incorporate drugs and proteins into chylomicrons through the agency of a liposome-like vesicle composed of cholesterol esters.

##### Endocytotic Pathway

A formulation developed by Diabetology Ltd. is a combination of a natural bile salt and other GRAS-listed excipients which exert three different activities to enhance passage of biologics across the intestinal wall—(i) a transient protease inhibitory activity to prevent breakdown of formulated biologics in the lumen of the gut, (ii) a mucolytic action to increase access to cells across the mucin layer, and (iii) a cell stimulatory activity resulting in uptake of proteins into cells via a fluid-phase vacuolation process. This uptake mechanism is one which occurs naturally as a means of absorption of digested nutrients; it is usually quiescent in the fasted state, but can be up-regulated by the formulation excipients to allow ingestion of intact proteins which have escaped protease attack.

The formulation is a dry powder, administered in an enteric-coated capsule, which breaks down in the duodenum to release its contents which dissolve rapidly in the aqueous lumenal fluid. Preclinical studies have shown levels of protein in peripheral blood consistent with a ~10% bioavailability, with significantly higher concentrations in the portal vein, confirming that as the point of entry into the body. A clamp study of oral insulin in type 2 volunteers has shown effects on glucose disposal lasting for 9–14 h. [70]. In these experiments, peripheral insulin levels did not rise above baseline, indicating that all of the insulin was absorbed by the liver before reaching the outer circulation. The formulation is stable at room temperature for long periods of time, and no hypos have been observed in patients treated. The product is currently in phase 2b clinical trials.

### 3.3. Tissue-Specific Approaches (Physical)

A means of administering a protein by injection, but from within the small intestine itself, has recently been developed by Rani Therapeutics. It consists of an enteric-coated capsule which conceals a needle capable of penetrating the intestinal wall [https://www.ranitherapeutics.com/technology/]. A key requirement is to make sure that the wall of the capsule is as close as possible to the wall of the intestine, and this objective is achieved by arranging that carbon dioxide is generated inside the capsule as soon as the enteric coat dissolves, which causes the capsule to swell to fill the whole of the lumen of the gut. At the same time, a dissolvable needle pierces the capsule shell and enters the gut wall, depositing a solid plug of peptide close to the intestinal microvasculature, where it can dissolve and enter the circulation.

The capsule itself is insoluble, and voided in the stool, while the bioavailability of the biologic is said to be 70% or more. A phase 1 study has indicated no safety issues. The capsule has the potential to communicate with digital apps once delivery has been registered, in order to check on patient compliance, etc. At the present time, there are no data confirming the reproducibility of the device. While the capsule is ambitious in its concept, and a miracle of engineering, one wonders whether its size and complexity may cause problems. It appears to have the dimensions of a size 0 capsule, and safety concerns need to be allayed for an approach which essentially works by causing an intestinal obstruction. It also remains to be seen whether its high delivery capability will offset the manufacturing costs involved in putting together all the components required for reliable operation.

## 4. Conclusions

Oral delivery of large peptides and proteins is an endeavour which has come of age. With a number of different clinical trials showing success in terms of efficacy, there will surely be products on the market in the next few years, and the factors limiting their appearance will not be technical, so much as economic and logistic. Costs for large-scale manufacture of biologics have come down significantly in the last few years; the fact that only a portion of the payload is delivered across the gut is no longer a problem, and biopotency levels of 10% are sufficient to yield products that are highly viable from an economic standpoint. While a bane from the delivery point of view, the high proteolytic activity in the gut means that any material that is not taken up is rapidly broken down into non-toxic components, and so no safety issues are raised. The shift of emphasis thus moves to the nature of the vehicle, which may often outweigh the API by a factor of ten to one thousand. Excipients that are cheap, GRAS-listed or pharmacopoeial, stable and have a good safety record are clearly advantageous. Biologics already play an important role in the treatment of disease, in areas such as diabetes (insulin, GLP-1) osteoporosis (sCT and PTH) and rheumatoid arthritis (TNF blockade) and treatment of these, and other chronic disorders, will soon be transformed by the advent of the up-coming methods described here for their delivery as oral medications.

## Figures and Tables

**Table 1:** Properties of intestinal enzymes.

Enzyme	Specificity	pH Optimum	Classification	Inhibitors
Trypsin	Lys, Arg	7.5–8.5	Serine protease	Aprotinin, SBTI, DFP
Chymotrypsin	Tyr, Phe, Trp	7.8–8.0	Serine protease	Coumarin, boronic acid, hydroxymethyl pyrrole
Carboxypeptidase A	Aromatic or branched sidechains	7–9	Metallo-protease (Zn atom)	Metal ions, 1,10-phenanthroline, ochratoxin A, sulphides
Carboxypeptidase B	Arg, Lys, also Val, Ile, Asn Gly, Gln	9.0	Metallo-protease (Zn atom)	Heavy metals, 1,10-phenanthroline, EDTA. Arginine, lysine, ornithine, inhibitors from potato and leech
Elastase I	Ala-Ala, Ala-Gly	8.5	Serine protease	Serpins, dipeptides of Ala, Val, Leu and Ile
Elastase II	Medium to large hydrophobic residues			

Data drawn principally from the Worthington Enzyme Manual [http://www.worthington-biochem.com/].
